# Supplementary material for: MetaRibo-Seq measures translation in microbiomes
Source: Nat Commun. 2020 Jun 29;11:3268. doi: 10.1038/s41467-020-17081-z (PMC7324362; doi:10.1038/s41467-020-17081-z)
Supplement: Supplementary file 10 — Supplementary Data 7 [file 41467_2020_17081_MOESM10_ESM.zip › File2/Confidence_High_Taxonomy/435874_out.krona.html]

Javascript must be enabled to view this page.

members
magnitude
magnitudeUnassigned
count
unassigned
taxon
rank

435874\_out

337

superkingdom
337
2

332
976
phylum

class
200643
332

171549
332
order

family
1
2005525

1
375288
genus

1
1750560
species

SRS098717\_contig\_number\_23494

3
171552
family

1
577309
genus

species

SRS063370\_contig\_number\_9782
454154
1

genus
2
838

2
59823
species

SRS045713\_contig\_number\_16163SRS048164\_contig\_number\_15308

family
1
2005519

1348911
1
genus


SRS014923\_contig\_number\_37288
species
1
1941478

327
815
family

327
816
40

SRS011302\_contig\_number\_30759SRS013638\_contig\_number\_15117SRS014287\_contig\_number\_16259SRS015369\_contig\_number\_7907SRS015578\_contig\_number\_contig-100\_176.203112SRS015794\_contig\_number\_4931SRS015854\_contig\_number\_22618SRS015890\_contig\_number\_12136SRS016095\_contig\_number\_28096SRS016203\_contig\_number\_contig-100\_525.69803SRS017821\_contig\_number\_30144SRS018133\_contig\_number\_11557SRS019496\_contig\_number\_16506SRS020328\_contig\_number\_38926SRS022071\_contig\_number\_33488SRS023526\_contig\_number\_24582SRS023583\_contig\_number\_2840SRS023971\_contig\_number\_3067SRS043411\_contig\_number\_contig-100\_1367.104566SRS046717\_contig\_number\_8877SRS049896\_contig\_number\_26555SRS050299\_contig\_number\_13874SRS050925\_contig\_number\_17285SRS051610\_contig\_number\_contig-100\_1173.74024SRS052697\_contig\_number\_38929SRS064557\_contig\_number\_1877SRS074586\_contig\_number\_2486SRS075963\_contig\_number\_contig-100\_268.268SRS077294\_contig\_number\_12877SRS100907\_contig\_number\_4319SRS1041140\_contig\_number\_13636SRS104400\_contig\_number\_52815SRS143722\_contig\_number\_contig-100\_435.89746SRS144537\_contig\_number\_48111SRS147139\_contig\_number\_9325SRS150029\_contig\_number\_10128SRS892882\_contig\_number\_4953SRS893366\_contig\_number\_18954SRS893378\_contig\_number\_contig-100\_95.127470SRS893380\_contig\_number\_contig-100\_335.31598
genus

2292956
1
species

SRS024132\_contig\_number\_12420


SRS143148\_contig\_number\_3802
species
1262736
1

2292002
1

SRS012273\_contig\_number\_22569
species


SRS077194\_contig\_number\_1790SRS1041142\_contig\_number\_1364
species
818
2

species

SRS014459\_contig\_number\_34733SRS049446\_contig\_number\_7389SRS062701\_contig\_number\_5938SRS893373\_contig\_number\_6231
4
246787

species

SRS1041145\_contig\_number\_26771
1
2302926

species

SRS011239\_contig\_number\_20777
1
310298

species

SRS011134\_contig\_number\_22714SRS011405\_contig\_number\_15186SRS012849\_contig\_number\_contig-100\_1772.207183SRS012969\_contig\_number\_30933SRS013098\_contig\_number\_12296SRS013476\_contig\_number\_28022SRS013521\_contig\_number\_2253SRS013687\_contig\_number\_29798SRS013800\_contig\_number\_23817SRS013940\_contig\_number\_6431SRS013951\_contig\_number\_33754SRS014235\_contig\_number\_28751SRS014313\_contig\_number\_3984SRS014613\_contig\_number\_6460SRS014683\_contig\_number\_4016SRS014948\_contig\_number\_20304SRS014979\_contig\_number\_3716SRS015065\_contig\_number\_31046SRS015264\_contig\_number\_1978SRS015431\_contig\_number\_35212SRS015579\_contig\_number\_14075SRS015663\_contig\_number\_36054SRS015694\_contig\_number\_21924SRS016018\_contig\_number\_16321SRS016056\_contig\_number\_6037SRS016335\_contig\_number\_48429SRS016495\_contig\_number\_12489SRS016840\_contig\_number\_2194SRS016897\_contig\_number\_871SRS016989\_contig\_number\_7092SRS017103\_contig\_number\_16823SRS017247\_contig\_number\_6563SRS017307\_contig\_number\_14369SRS017433\_contig\_number\_6167SRS017521\_contig\_number\_107SRS017701\_contig\_number\_10656SRS017745\_contig\_number\_2390SRS018351\_contig\_number\_18098SRS018427\_contig\_number\_15366SRS018541\_contig\_number\_9607SRS018575\_contig\_number\_7349SRS018623\_contig\_number\_7452SRS018836\_contig\_number\_3466SRS018888\_contig\_number\_3685SRS018936\_contig\_number\_8670SRS018984\_contig\_number\_14269SRS019267\_contig\_number\_9258SRS019397\_contig\_number\_961SRS019445\_contig\_number\_7279SRS019601\_contig\_number\_19019SRS019685\_contig\_number\_27887SRS019693\_contig\_number\_1533SRS019808\_contig\_number\_19964SRS019910\_contig\_number\_contig-100\_312.136494SRS020271\_contig\_number\_268SRS020394\_contig\_number\_9694SRS020622\_contig\_number\_4336SRS021153\_contig\_number\_4374SRS021219\_contig\_number\_5914SRS021484\_contig\_number\_13609SRS021948\_contig\_number\_23134SRS022524\_contig\_number\_9516SRS022609\_contig\_number\_4882SRS023914\_contig\_number\_22851SRS024009\_contig\_number\_8376SRS024075\_contig\_number\_5367SRS024331\_contig\_number\_8969SRS024435\_contig\_number\_37061SRS024625\_contig\_number\_9437SRS042284\_contig\_number\_1476SRS042628\_contig\_number\_21465SRS043768\_contig\_number\_29615SRS045244\_contig\_number\_3323SRS045645\_contig\_number\_21838SRS045826\_contig\_number\_20520SRS046068\_contig\_number\_2287SRS046502\_contig\_number\_4839SRS047014\_contig\_number\_32666SRS048060\_contig\_number\_5124SRS048870\_contig\_number\_4008SRS048981\_contig\_number\_7061SRS049402\_contig\_number\_15113SRS050422\_contig\_number\_36001SRS050520\_contig\_number\_529SRS050801\_contig\_number\_1718SRS050941\_contig\_number\_14285SRS050974\_contig\_number\_24SRS050998\_contig\_number\_11718SRS051031\_contig\_number\_17951SRS051882\_contig\_number\_22615SRS052027\_contig\_number\_17639SRS052078\_contig\_number\_339SRS053214\_contig\_number\_21359SRS053335\_contig\_number\_7931SRS053398\_contig\_number\_4644SRS053649\_contig\_number\_11656SRS054956\_contig\_number\_contig-100\_167.96821SRS055017\_contig\_number\_16239SRS055982\_contig\_number\_11516SRS056273\_contig\_number\_11599SRS056519\_contig\_number\_10556SRS056537\_contig\_number\_38055SRS057478\_contig\_number\_2455SRS057480\_contig\_number\_3065SRS058723\_contig\_number\_6894SRS058770\_contig\_number\_23311SRS062427\_contig\_number\_10561SRS062654\_contig\_number\_26683SRS063518\_contig\_number\_31228SRS064276\_contig\_number\_27599SRS064645\_contig\_number\_2038SRS064757\_contig\_number\_22898SRS064973\_contig\_number\_1634SRS065176\_contig\_number\_15902SRS075021\_contig\_number\_6209SRS075078\_contig\_number\_20686SRS075716\_contig\_number\_3319SRS075821\_contig\_number\_630SRS075878\_contig\_number\_12347SRS075984\_contig\_number\_12980SRS076804\_contig\_number\_20311SRS076976\_contig\_number\_12897SRS077024\_contig\_number\_3126SRS077086\_contig\_number\_7452SRS077127\_contig\_number\_13141SRS077231\_contig\_number\_18847SRS077335\_contig\_number\_2278SRS077502\_contig\_number\_12630SRS077552\_contig\_number\_6491SRS077589\_contig\_number\_7967SRS077641\_contig\_number\_21655SRS077730\_contig\_number\_contig-100\_565.112026SRS078176\_contig\_number\_7769SRS078242\_contig\_number\_20488SRS078665\_contig\_number\_2391SRS097958\_contig\_number\_13718SRS098061\_contig\_number\_11857SRS098073\_contig\_number\_17632SRS098571\_contig\_number\_16571SRS098644\_contig\_number\_10369SRS100021\_contig\_number\_9327SRS101268\_contig\_number\_131SRS101376\_contig\_number\_13695SRS101433\_contig\_number\_7307SRS104084\_contig\_number\_11215SRS1041031\_contig\_number\_21204SRS1041032\_contig\_number\_16163SRS1041033\_contig\_number\_23780SRS1041037\_contig\_number\_14033SRS1041039\_contig\_number\_20428SRS1041091\_contig\_number\_18495SRS1041112\_contig\_number\_5936SRS1041129\_contig\_number\_11342SRS1041130\_contig\_number\_1417SRS1041133\_contig\_number\_20570SRS1041134\_contig\_number\_565SRS1041139\_contig\_number\_contig-100\_794.795SRS1041141\_contig\_number\_10642SRS1041143\_contig\_number\_3637SRS1041157\_contig\_number\_6975SRS1041164\_contig\_number\_2176SRS104311\_contig\_number\_10799SRS104975\_contig\_number\_2804SRS1054691\_contig\_number\_13668SRS1054716\_contig\_number\_contig-100\_499.85731SRS1055022\_contig\_number\_12977SRS1055038\_contig\_number\_3460SRS1055043\_contig\_number\_10255SRS1055049\_contig\_number\_11254SRS1055069\_contig\_number\_10125SRS1055076\_contig\_number\_16927SRS1055083\_contig\_number\_1362SRS1055099\_contig\_number\_7929SRS140492\_contig\_number\_12857SRS140513\_contig\_number\_9724SRS142503\_contig\_number\_24790SRS142542\_contig\_number\_7335SRS142599\_contig\_number\_24169SRS142781\_contig\_number\_11521SRS142890\_contig\_number\_25146SRS142923\_contig\_number\_15045SRS143085\_contig\_number\_553SRS143181\_contig\_number\_10400SRS143342\_contig\_number\_2511SRS143466\_contig\_number\_26482SRS143523\_contig\_number\_16102SRS143895\_contig\_number\_32242SRS144135\_contig\_number\_28726SRS144183\_contig\_number\_31068SRS144297\_contig\_number\_8264SRS144506\_contig\_number\_35140SRS144714\_contig\_number\_20435SRS145497\_contig\_number\_6438SRS146812\_contig\_number\_53289SRS147088\_contig\_number\_1861SRS147271\_contig\_number\_42441SRS147346\_contig\_number\_9625SRS147557\_contig\_number\_14616SRS147766\_contig\_number\_2642SRS147919\_contig\_number\_13353SRS147977\_contig\_number\_9327SRS148091\_contig\_number\_10678SRS148196\_contig\_number\_24847SRS148253\_contig\_number\_11071SRS148319\_contig\_number\_13077SRS148424\_contig\_number\_17695SRS148721\_contig\_number\_3147SRS148784\_contig\_number\_24573SRS148817\_contig\_number\_2128SRS148970\_contig\_number\_445SRS149181\_contig\_number\_30802SRS149325\_contig\_number\_12886SRS149784\_contig\_number\_14347SRS149879\_contig\_number\_1941SRS893172\_contig\_number\_14211SRS893187\_contig\_number\_17706SRS893230\_contig\_number\_8839SRS893231\_contig\_number\_1432SRS893253\_contig\_number\_462SRS893256\_contig\_number\_28SRS893270\_contig\_number\_2956SRS893279\_contig\_number\_4153SRS893292\_contig\_number\_3592SRS893300\_contig\_number\_contig-100\_1858.66128SRS893327\_contig\_number\_8874SRS893342\_contig\_number\_2770SRS893358\_contig\_number\_728SRS893369\_contig\_number\_contig-100\_1678.78261SRS893382\_contig\_number\_2255SRS893383\_contig\_number\_37157SRS971275\_contig\_number\_22834SRS971276\_contig\_number\_25365
232
820

species

SRS017859\_contig\_number\_1942SRS019161\_contig\_number\_7218SRS022137\_contig\_number\_10821SRS022713\_contig\_number\_227SRS045004\_contig\_number\_13827SRS1041137\_contig\_number\_17406
46506
6

4
28116
species

SRS065504\_contig\_number\_35798SRS075773\_contig\_number\_18205SRS097920\_contig\_number\_18066SRS143876\_contig\_number\_contig-100\_81.232229


SRS019286\_contig\_number\_268SRS104636\_contig\_number\_24009
species
2292913
2


SRS063928\_contig\_number\_345SRS149244\_contig\_number\_10643
species
1263055
2

4
47678
species

SRS019030\_contig\_number\_4851SRS020233\_contig\_number\_47150SRS077849\_contig\_number\_19651SRS078419\_contig\_number\_20524

1
1917883
species

SRS1055067\_contig\_number\_12465


SRS144603\_contig\_number\_12332
species
1
674529

821
8
species

SRS013158\_contig\_number\_1562SRS014855\_contig\_number\_15547SRS054905\_contig\_number\_12900SRS076756\_contig\_number\_13435SRS098655\_contig\_number\_contig-100\_81.80375SRS104327\_contig\_number\_1785SRS149075\_contig\_number\_15198SRS893295\_contig\_number\_9295


SRS049900\_contig\_number\_18733SRS147039\_contig\_number\_356
species
457393
2

1946089
8
species

SRS064232\_contig\_number\_2176SRS077392\_contig\_number\_22358SRS077454\_contig\_number\_12746SRS1041116\_contig\_number\_8718SRS104693\_contig\_number\_4557SRS1055095\_contig\_number\_380SRS147653\_contig\_number\_770SRS893252\_contig\_number\_17747

species

SRS1041138\_contig\_number\_10211
1
2292944

2292932
1

SRS144362\_contig\_number\_20397
species

817
1

SRS015190\_contig\_number\_contig-100\_549.101482
species


SRS143372\_contig\_number\_12071
species
1
1262735

species

SRS103987\_contig\_number\_26399
1
371601

species

SRS023715\_contig\_number\_20259
1
2292924

1239
5
phylum

186801
5
class

186802
5
order

541000
2
family

1
1263
genus

1
41978

SRS016954\_contig\_number\_4041
species

1
946234
genus

1
292800
species

SRS011084\_contig\_number\_23232

family
186803
2

2
39491
species

SRS019068\_contig\_number\_18762SRS143991\_contig\_number\_18053

family
31979
1

genus
1485
1


SRS049995\_contig\_number\_25299
species
1262793
1
